# *FMR1* Low Zone CGG Repeats: Phenotypic Associations in the Context of Parenting Stress

**DOI:** 10.3389/fped.2020.00223

**Published:** 2020-05-14

**Authors:** Marsha R. Mailick, Jinkuk Hong, Leann Smith DaWalt, Jan S. Greenberg, Arezoo Movaghar, Mei Wang Baker, Paul J. Rathouz, Murray H. Brilliant

**Affiliations:** ^1^Waisman Center, University of Wisconsin-Madison, Madison, WI, United States; ^2^Wisconsin State Laboratory of Hygiene, Madison, WI, United States; ^3^Dell Medical School at the University of Texas at Austin, Austin, TX, United States; ^4^Marshfield Clinic Research Institute, Marshfield, WI, United States

**Keywords:** *FMR1* CGG repeats, gene x environment interactions, stressful parenting, trinucleotide repeat disorders, low zone genotype-phenotype associations

## Abstract

The *FMR1* gene on the X chromosome has varying numbers of CGG repeats. The modal number is 30, and expansion to >200 results in fragile X syndrome, but the copy number extends down to 6. Past research suggests that individuals whose CGGs are in the “low zone” (LZ; defined here as ≤ 25 CGGs) may be more environmentally-reactive than those with normal range repeats (26–40 CGGs)—a gene x environment interaction. Using a population-based DNA biobank, in our primary analysis we compared 96 mothers with LZ CGG repeats on both alleles to 280 mothers who had CGG repeats in the normal range. Secondarily, we conducted parallel analyses on fathers. We investigated how parents in these two CGG repeat categories differentially responded to stress, defined as parenting a child with disabilities. Significant gene x environment interactions indicated that LZ mothers who had children with disabilities had greater limitations (in executive functioning, depression, anxiety, daily health symptoms, and balance) than LZ mothers whose children did not have disabilities. In contrast, mothers with normal-range CGG repeats did not differ based on stress exposure. For fathers, a similar pattern was evident for one phenotype only (hand tremors). Although on average LZ CGGs are not associated with compromised functioning, the average masks differential response to the environment.

## Introduction

The *FMR1* gene on the X chromosome encodes the fragile X mental retardation protein (FMRP), an RNA-binding protein that regulates the expression of hundreds of genes and plays a key role in brain development and function (1, 2). In the 5′ untranslated region of the *FMR1* mRNA, there are varying numbers of CGG trinucleotide repeats. The modal number of CGG repeats in the human population is around 30, whereas expansion above 200 CGG repeats leads to fragile X syndrome (FXS). CGG repeats between 55 and 200 are classified as “premutation,” because individuals with CGGs in this range are at increased risk for having children with full mutation FXS, and may also be at higher risk themselves for motor, reproductive, and mental health symptoms (3–5). Those in the “gray zone” (variously defined as 45–54 or 41–54 CGG repeats) are at risk for repeat instability and expansion when passed to subsequent generations, and there is emerging evidence that a small sub-group of those in the gray zone may have elevated risks of motor (6, 7) and reproductive (8) symptoms.

The focus of this study is on *FMR1* CGG repeats *below the gray zone*, considered normal by the American College of Medical Genetics and Genomics [ACMG; (9)]. Even in the putative normal range, the number of *FMR1* CGG repeats in the human population is highly polymorphic (10, 11) and the reported copy number extends down to 6 CGGs (10, 12–14).

Given this level of polymorphism, much may be learned by exploring phenotypic associations of low copy numbers of CGG repeats within *FMR1*.

## Genotype-Phenotype Associations

Several previous reports probed genotype-phenotype associations across the full *FMR1* CGG repeat range (below the FXS level of 200+ CGG repeats). These reports suggest that the phenotypes associated with both lower than normal and expanded numbers of repeats might be *symmetrical*. Chen et al. (13) transfected synthetic human *FMR1* promoter sequences into cell lines, and showed that there was reduced protein translation at both low and high numbers of CGG repeats compared to 30 CGGs. Nagamani et al. (15), in case reports, showed that duplication and deletion of *FMR1* can lead to overlapping clinical neurodevelopmental phenotypes. Ramocki and Zoghbi (16) argued that there is a need for tight neuronal homeostatic control mechanisms for normal cognition and behavior, and that imbalances in homeostatic controls in multiple genes, including *FMR1*, might be responsible for neurodevelopmental and neuropsychiatric disorders. Together these reports suggest that both low and high numbers of CGG repeats in *FMR1* result in similar neurodevelopmental phenotypes.

Motivated by this suggestion of symmetry in the phenotypes associated with both low and expanded copy numbers of *FMR1* CGG repeats, our group previously used representative population survey data from the Wisconsin Longitudinal Study (WLS) to examine genotype-phenotype associations in a birth cohort of aging adults (*n* = 6,747; mean age = 71) who had CGG repeats ranging from 8 to 134. Using this data, we explored the interaction between the number of CGG repeats and stress exposure, defined as parenting an adult son or daughter who had mental health problems or developmental disabilities (17). Among parents of adults with such disabilities, having CGG repeats either in the low or expanded range was associated with *worse* health and functional limitations than having average numbers of CGG repeats. In contrast, among those who did not have children with such disabilities, having CGG repeats either in the low or expanded range was associated with *better* health and functional outcomes than those having average numbers of CGG repeats.

This pattern of gene by environment interactions was interpreted to be consistent with “differential susceptibility” (18, 19) or the “flip-flop phenomenon” (20, 21). Both of these conceptualizations, though drawing from different research literatures, hypothesize that people with certain genotypes are more reactive to the environment—both positive and negative environments—whereas those who have other genotypes are less environmentally-reactive.

## The Present Study

Given contemporary concerns about reproducibility of genetic results (22), there is a need to verify novel findings. The present study aimed to both replicate and also to extend our prior WLS research using data from a completely independent population. As the present study drew from a population nearly three times larger than the WLS sample, we were able to design this new analysis to control for the influence of factors that might otherwise confound the effect of low numbers of CGG repeats.

Specifically, although our past WLS research did not separately analyze data from men and women, we were able to do so in the present study. This was particularly advantageous given our focus on stressful parenting, as research has shown that family caregiving often has greater impacts on mothers than fathers [e.g., (23–28)]. We further restricted our sample of mothers in the present analysis to those who were homozygous for CGG repeats, which we defined as having both alleles within the low range of the CGG distribution or both alleles within the normal range of CGG repeats (see below for definitions of low and normal-range CGGs). In contrast, in our previous WLS research on CGG effects and stressful parenting, we included both homozygous and heterozygous women. Additionally, the population studied in our prior WLS research was based on members of a single birth cohort and their siblings. The members of that birth cohort were age 71 at the time the relevant data were collected. In contrast, the participants in the present study ranged in age from 28 to over 90, making it possible to explore here how age may interact with stressful parenting and CGG effects. However, unlike the WLS research, the present study was not able to investigate the effect of CGG *expansions* because there were no women (out of 11,526) who were homozygous in the premutation range and very few men with premutation alleles (*n* = 27 out 8,463).

The major hypothesis of this study is that, for women, having low vs. normal numbers of CGG repeats in *FMR1* interacts with stress exposure to predict divergent profiles in specific cognitive, mental health, and physical health phenotypes. The focus on these phenotypes was motivated by prior reports of symmetry in low and expanded copy numbers of CGG repeats in *FMR1* vs. modal copy numbers; all of these phenotypes have been shown to be associated with expanded numbers of CGG repeats [e.g., (5)]. The specific phenotypes include executive functioning, depressive symptoms, anxiety symptoms, daily health symptoms, problems with balance, hand tremors, and for women age at menopause and severity of menopause symptoms. We hypothesized that there would be a significant interaction between stressful parenting status and CGG repeat category, such that among mothers in the low zone, those whose children have disabilities will have *poorer* outcomes for these phenotypes than those whose children are not disabled, whereas among mothers with normal-range CGGs, those who have children with disabilities will not differ from those whose children are not disabled. Our major focus is on mothers because, as noted, the findings of past research showed greater evidence for parenting stress effects for mothers than for fathers. However, we also explored whether these same patterns would be evident for fathers.

As a follow-up analysis, for those phenotypes for which the predicted gene x environment interaction effects are found to be significant, we explore age effects. We pursue this exploratory analysis because exposure to parenting stress likely varies throughout the parent's life course. Although parenting is a lifelong role, and parenting adult children with disabilities remains stressful even after the children reach adulthood (29), the intensity of parenting is greatest in its early years. Therefore, here we explore whether the hypothesized gene x environment interaction differs by parental age.

## Methods

### Population

The study sample was drawn from the population-based Marshfield Clinic Personalized Medicine Research Project (PRMP). Starting in 2002, PMRP enrolled ~20,000 individuals (40% of the eligible population of the Marshfield Epidemiologic Study Area, a 19-zipcode region centered geographically around Marshfield Wisconsin and an additional 9-zipcode area in northern Wisconsin) who consented to share their DNA and participate in research (30). The participants were diverse in age, with birth years ranging from pre-1922 to 1991. The overwhelming majority of PMRP members are White non-Hispanic (98.4%). This research was approved by the Institutional Review Boards of the Marshfield Clinic Research Institute and the University of Wisconsin-Madison. All participants provided written informed consent prior to their inclusion in the study.

### Definition of Low and Normal-Range CGG Repeats

Precisely defining the categories that constitute the *FMR1* CGG repeat range is challenging due to both scientific and technical factors. The published guidelines provided by the American College of Medical Genetics and Genomics for defining normal and mutation categories in *FMR1* (9) note that the borders of the various categories are approximate. “Each definition may change with increased empirical data and research” (p. 578), and there is an acceptable margin of error of several CGG repeats at the borders of the categories.

In our earlier descriptive study using the WLS (31), we defined the “low zone” statistically (≤23 CGGs, which was 2 SDs below the mean of the WLS distribution). We adopted this approach for that descriptive study because extant research did not offer a biological or clinical basis to differentiate normal-range from low numbers of CGG repeats. However, one study (32) defined low numbers of CGGs as ≤25 repeats. Recognizing the margin of error described by the ACMG, we adopted the more inclusive definition of the low zone for the present analysis (i.e., ≤25 repeats).

Definition of normal-range CGGs also has varied in past research. Weghofer et al. (32) defined the normal range narrowly as 26 to 34 CGGs. The ACMG defines normal as any number of CGGs below the gray zone, i.e., <45 CGGs. Some researchers have defined normal as <41 CGGs based on the downward extension of the gray zone to 41 CGG (7, 33–35), referred to in Maenner et al. (36) as the “expanded gray zone.” Therefore, for the present research, we defined the normal range as 26 to 40 CGG repeats (i.e., beginning one CGG repeat above the upper bound of the low zone and ending one CGG repeat below the lower bound of the expanded gray zone).

### Data

**Figure 1:**
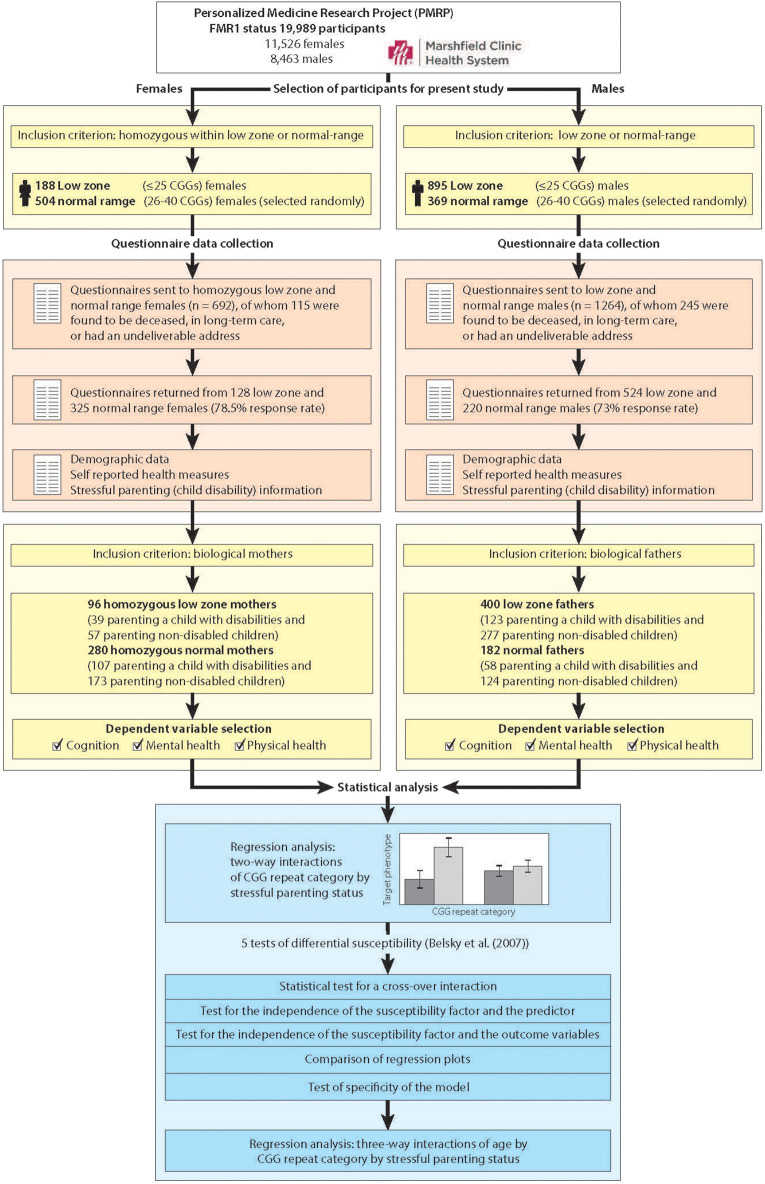
Workflow overview of selection of participants, data collection, and statistical analysis.

Figure 1 portrays the study workflow, including genetic screening of the population, selection of participants who met inclusion criteria, data collection, and statistical analysis. We show separate workflows for females and males because of the added step of needing to identify females who were homozygous within the low zone or the normal-range.

Using procedures described previously (36, 37), 19,989 DNA samples from PRMP participants were assayed for *FMR1* CGG repeat length, of which 11,526 were samples from females and 8,463 were samples from males. We identified 188 females who had low zone CGG repeats on both alleles, and 895 males with CGG repeats in the low zone. We randomly selected 873 controls with normal-range CGG repeats (504 homozygous females and 369 males). The number of randomly selected normal-range females and males was based on a power analysis designed to yield 80–90% power to detect small (0.2–0.4) Cohen's D phenotypic effect sizes between groups (38).

As part of a larger study, questionnaires were sent to these 1956 men and women, of whom 360 were subsequently found to be deceased, in long-term care, or had an undeliverable address at the time of the questionnaire survey (*n* = 115 females and 245 males). Of the remaining potential participants, 78.5% of the females and 73.0% of the males completed and returned questionnaires. The questionnaires encompassed a broad range of demographic and phenotypic data, including measures of cognitive functioning, mental health and physical health (described below). Also measured were characteristics of participants' children, including child disabilities, the variable that was used in the identification of stressful parenting status. (Note that when we refer to the children of these parents, we are referring to sons and daughters of any age, including adult children).

An additional inclusion criterion for the present analysis was parental status, since a primary focus of the present study is on exposure to stressful parenting. Thus, the present analytic sample included 96 mothers who were homozygous for CGG repeats within the low zone (≤25 repeats) and 280 mothers who were homozygous for CGG repeats in the normal range (26–40 repeats), for a total analytic sample size of 376 mothers. The analytic sample also included 400 fathers in the low zone and 182 who had normal-range CGG repeats. None of the participants were aware of their *FMR1* status (per IRB stipulation).

### Definition of Stressful Parenting

Parents reported whether any of their biological children had a developmental (e.g., ADD/ADHD, learning disability, autism spectrum disorder) or a mental health (e.g., anxiety/depression, bipolar disorder, alcohol/drug problems) condition, and if so they were included in the “stressful parenting” group. The comparison group consisted of parents who did not report that any of their children had a developmental or mental health condition.

To confirm that having a child with disabilities is a valid indicator of stressful parenting, we compared parents who had a child with a disability and parents who did not have any children with disabilities with respect to objective and subjective indicators of parenting stress. For the *objective measure*, we calculated the number of negative life events that parents reported were experienced by their children during the past year, derived from Abidin's (39) Life Stress Scale of the Parenting Stress Index. Parents of children with disabilities reported that their children experienced significantly more negative life events during the past year than parents of children who did not have disabilities (*F* = 10.05, *p* < 0.01 and *F* = 13.10, *p* < 0.001 for mothers and fathers, respectively). For the *subjective measure*, we used the Parenting Stress Scale (40), a self-report 18-item measure with previously established reliability and validity. In the present study, parents of children with disabilities reported significantly higher levels of parenting stress than parents whose children did not have disabilities (*F* = 39.8, *p* < 0.001, and *F* = 33.8, *p* < 0.001 for mothers and fathers, respectively).

These descriptive comparisons confirm that the two parenting groups—those who had a child with disabilities and those whose children did not have disabilities—differed in parenting stress, objectively and subjectively measured. Notably, there were no significant differences in these stress markers between parents with CGGs in the low zone and those with normal-range CGGs.

### Phenotypic Measures

#### Cognition

To measure cognitive functioning, we administered the BRIEF-A [Behavior Rating Inventory of Executive Functioning—Adult Version, (41)], a standardized self-report measure that captures an adult's executive functions or self-regulation in the everyday environment. The BRIEF-A includes 75 items within nine non-overlapping theoretically and empirically derived clinical scales: Inhibit, Self-Monitor, Plan/Organize, Shift, Initiate, Task Monitor, Emotional Control, Working Memory, and Organization of Materials. Together, they constitute the BRIEF-A Global Executive Composite (GEC), which was the measure analyzed for the present study. For the normative sample (41), T-scores were normalized to have a mean of 50 and standard deviation of 10, and in the present sample, the mean was 50.5 with a standard deviation of 10.4. About 10 percent of the present sample (10.5% of mothers and 10.4% of fathers) had a T-score of 65 or higher, the suggested cutoff for clinically significant executive dysfunction. For the normative sample, Cronbach's alpha = 0.96. For the present sample, Cronbach's alpha was also 0.96 for both the mother sample and father samples.

#### Mental Health

Mental health was measured by two standardized self-report measures. Depressive symptoms were assessed by the Center for Epidemiological Studies-Depression Scale [CES-D; (42)]. For each of 20 depression symptoms, the respondent was asked to indicate how many days in the past week the symptom was experienced (0 = less than 1 day to 3 = 5 to 7 days). Examples of items included “I was bothered by things that usually don't bother me,” “I had trouble keeping my mind on what I was doing,” “I was depressed,” “I felt that everything I did was an effort.” The total score is the sum of the ratings for the 20 items, ranging from 0 to 60. Radloff (42) reported Cronbach's alpha to be 0.84–0.85. For the current analytic mother sample, Cronbach's alpha = 0.90, the mean was 8.9 (s.d. = 8.9), and 17% of the sample had scores 16 or higher, the cutoff for the clinical depression. For fathers, Cronbach's alpha = 0.86, the mean was 6.9 (s.d. = 7.3), and 10% of the sample had scores 16 or higher.

Anxiety was measured by the POMS (Profile of Mood States) Tension-Anxiety scale (43), which is a summary score of 9 items asking the degree to which respondents felt each of the following emotional states during the past week: tense, shaky, on edge, panicky, relaxed, uneasy, restless, nervous, and anxious (the positive item was reverse coded). Items were rated on a 5-point scale (0 = not at all to 4 = extremely) and summed, with the total score ranging from 0 to 36. In an adult sample, Cronbach's alpha was reported to be 0.89 (44). For the current analytic mother sample, Cronbach's alpha was also = 0.89. The sample mean of the Tension-Anxiety scale was 6.0 (s.d. = 5.7). For fathers, Cronbach's alpha = 0.84, and the mean was 4.9 (s.d. = 4.5).

#### Physical Health

Physical health was assessed by three indicators: a count of daily health symptoms, two items reflecting FXTAS-type motor symptoms, and two items reflecting FXPOI-type reproductive symptoms. To measure daily physical health symptoms, we used an adapted version of Larsen and Kasimatis' (45) physical symptom checklist, consisting of 27 health symptoms. For each symptom, the respondents indicated whether they experienced the symptom during the past 24 hours, and if affirmative, they reported the degree of the severity on a 10 point scale, 1 = very mild to 10 = very severe. The list included symptoms such as headache, backache, fatigue, joint pain, sore throat, cold/flu, nausea, and diarrhea. For the present measure, only symptoms with severity ratings of 4 or higher were selected, and the summary score was calculated by counting the number of symptoms that met this criterion. We selected the cut-off severity rating of 4 or higher because the average rating of symptoms (of those who reported any symptoms) was 3.6. Thus, a rating of 4 or higher represented the more severe range of the rating scale. For mothers, the average number of health symptoms above a severity rating of 4 was 2.2 (s.d. = 2.7), ranging from 0 to 17. For fathers, the average number of health symptoms above a severity rating of 4 was 1.6 (s.d. = 2.5), ranging from 0 to 15.

Two items were used to measure motor functioning; “Have you had any problems with your balance” and “Do you have tremor (shakiness) of your hands?” with a binary response, 0 = absent, 1 = present. About a quarter of the mothers (23.9%) and one-fifth of fathers (19.4%) reported having problems with balance, and just under one-tenth of the sample members (9.6% of mothers 8.8% of fathers) reported having hand tremors.

For mothers, two variables were used to measure reproductive phenotypes: the severity of menopausal symptoms and age at menopause. The severity of menopausal symptoms was measured for those who were peri-menopausal, menopausal, or post-menopausal (*n* = 307) using a summary score of 6 items rating the severity of menopausal symptoms (0 = not at all to 3 = a lot): hot flushes/flashes, depression, sleep disturbance, bone pains, night sweats, and other symptoms. For the current analytic mother sample, Cronbach's alpha = 0.72, and the mean was 5.6 (s.d. = 3.7) with scores ranging from 0 to 18. Mothers who were post-menopausal (*n* = 145) reported their age at last menstrual period (mean = 50.4, s.d. = 5.3), ranging from 37 to 60.

### Statistical Analysis

The data for mothers and fathers were analyzed separately. To test the hypothesis of gene x environment interactions, we estimated regression models with indicator variables for CGG repeat category (low zone vs. normal-range CGGs) and for stressful parenting status (parenting a child with disabilities vs. parenting non-disabled children) as the key predictors. The key variable of interest was the interaction of CGG repeat category by stressful parenting status. Model 1 is the main effects model, including two control variables (parental age and number of biological children) as well as variables for CGG repeat category and stressful parenting category. In Model 2, the interaction between CGG repeat category and stressful parenting category is added. If this interaction is non-significant, Model 1 results are interpreted.

In PMRP, it was possible for several members of a family to volunteer for and be included in the research. Although the number of such family members who met criteria for the present study was small, nevertheless to account for the potential dependency of observations, we used the Generalized Estimating Equation with the exchangeable correlation structure (GEE) (46) approach to regression modeling, and report regression coefficients with robust standard errors based on clustering at the level of family. The use of robust standard errors also protects against bias in inferences due to heteroskedasticity.

Next we tested for differential susceptibility by following the steps outlined in Belsky et al. (18). Step 1 involves a statistical test for a cross-over interaction. Step 2 involves testing the independence of the susceptibility factor (low zone vs. normal-range CGG repeats in the present study) and the predictor (stressful parenting status). Step 3 involves testing the independence of the susceptibility factor and the outcome variables. Step 4 involves comparison of the regression plot with prototypical displays in Figure 1 in Belsky et al. (18). Step 5 involves replacing the susceptibility factors and outcomes with different susceptibility factors and outcomes to test the specificity of the model. For Step 5, we demonstrated the specificity of the model with other outcomes. However, we did not have access to a different susceptibility factor within the PMRP population, so we could only partially test Step 5.

Finally, in exploratory analyses, given the large age range of mothers and fathers in the present sample, we probed whether variation in parental age further conditioned the variability in parenting effects. To do so, for those dependent variables that met the criteria for differential susceptibility, we examined the three-way interaction of gene (low zone vs. normal range) by environment (parenting a child with disabilities vs. parenting non-disabled children) by age (measured continuously). Because few members of the present sample were over the age of 85, for this step in the analysis the age variable was top-coded at 85.

## Results for Mothers

### Descriptive Findings

There were no significant differences between the mothers who had children with disabilities and those who did not with respect to CGG repeat number on either the shorter or longer allele. Consistent with population estimates (47, 48), more than a third of the mothers in the present sample (*n* = 146, 38.8%) had at least one child with the range of disabilities included here (see Table 1 for a listing of these diagnoses). Mothers in the low zone and mothers with normal-range CGGs did not differ in their likelihood of having children with disabilities (40.6 vs. 38.2%, respectively), nor did they differ in the specific diagnosis of their child. As shown in Table 1, the most common conditions affecting the children in both CGG groups were anxiety/depression (*n* = 58) and ADD/ADHD (*n* = 35). Very few had a child with severe conditions such as schizophrenia or autism spectrum disorder.

**Table 1:** Developmental or mental health conditions of mothers' biological children.

**Condition**	**Frequency**	**Percentage (%)**
None	230	61.17%
Anxiety/depression	58	15.43%
ADHD	35	9.31%
Autism spectrum disorders	13	3.46%
Developmental disabilities^a^	12	3.19%
Seizures	7	1.86%
Learning disabilities	5	1.33%
Alcohol/drug problems	5	1.33%
Bipolar disorder	5	1.33%
Schizophrenia	3	0.01%
Other^b^	3	0.01%
Total	376	100.0%

a *Including cerebral palsy, Down syndrome, intellectual disabilities, Tourette syndrome*.

b *Including sensory loss, mild mental health conditions*.

*Some mothers in the present study had more than one child with a developmental or mental health condition. The conditions of the children within each family were independently reviewed by three experienced raters (authors LSD, MM, JH) and the condition determined to be most severe was reported here in this table*.

Table 2 presents the characteristics of mothers divided into four groups based on CGG repeat category and stressful parenting. The four groups did not differ with respect to level of education, current marital status, current employment status, or household income. About half of the mothers had at least some post-high school education and were currently employed. The average household income was ~$55,000. Nearly one-quarter (23.6%) had annual household incomes of $30,000 or less, reflecting the economic diversity of the participants in the present study (data not shown).

**Table 2:** Demographic characteristics of mothers by CGG category and stressful parenting status.

	**Low zone**		**Normal-range**		
	**Children without disabilities (*n* = 57)**	**Children with disabilities (*n* = 39)**	**Children without disabilities (*n* = 173)**	**Children with disabilities (*n* = 107)**	**Total (*n* = 376)**
Age	55.9 (18.1) [29, 91]	53.8 (16.0) [30, 98]	60.0 (16.4) [28, 95]	58.6 (14.0) [29, 91]	58.3 (16.1) [28, 98]
Some post-high school education	53.5%	56.4%	52.3%	61.7%	55.6%
Currently married	80.3%	59.0%	74.2%	72.6%	73.1%
Household income	$58K	$52K	$54K	$55K	$54K
Currently employed	58.9%	66.7%	53.4%	56.0%	56.4%
Number of biological children	3.1 (1.6) [1,7]	2.9 (1.3) [1,6]	2.8 (1.5) [1,8]	2.8 (1.4) [1,8]	2.9 (1.5) [1,8]

However, there were significant differences with respect to maternal age and number of biological children. Mothers in the low zone were significantly younger on average than mothers with normal-range CGGs (55.5 vs. 59.4 years of age) and had a significantly greater number of biological children (3.2 vs. 2.9, on average). Notably, both of these variables had large ranges (maternal age ranged from ~30 to over 90 years of age in each group, and the number of biological children ranged from one to eight). Therefore, in all analyses, maternal age and number of biological children were statistically controlled.

### Multivariate Findings

**Figure 2:**
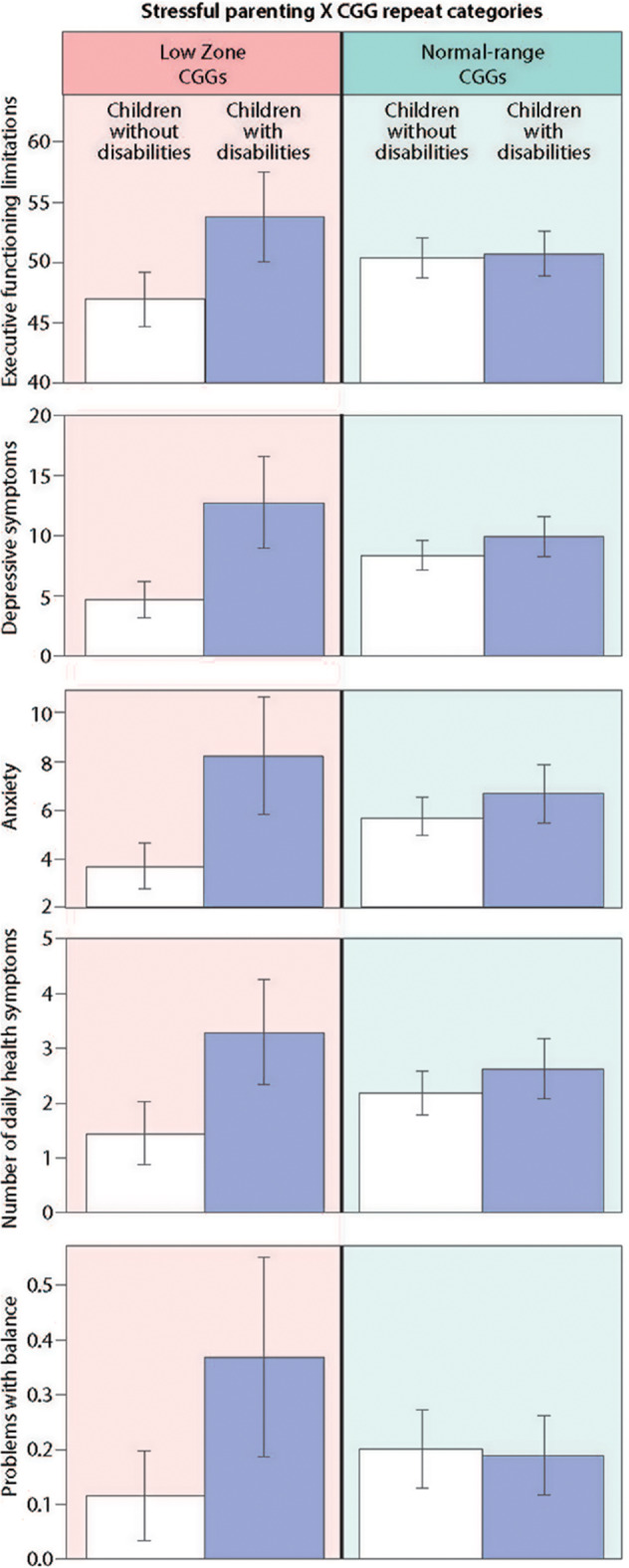
Significant interaction effects of stressful parenting status by CGG repeat category.

**Figure 3:**
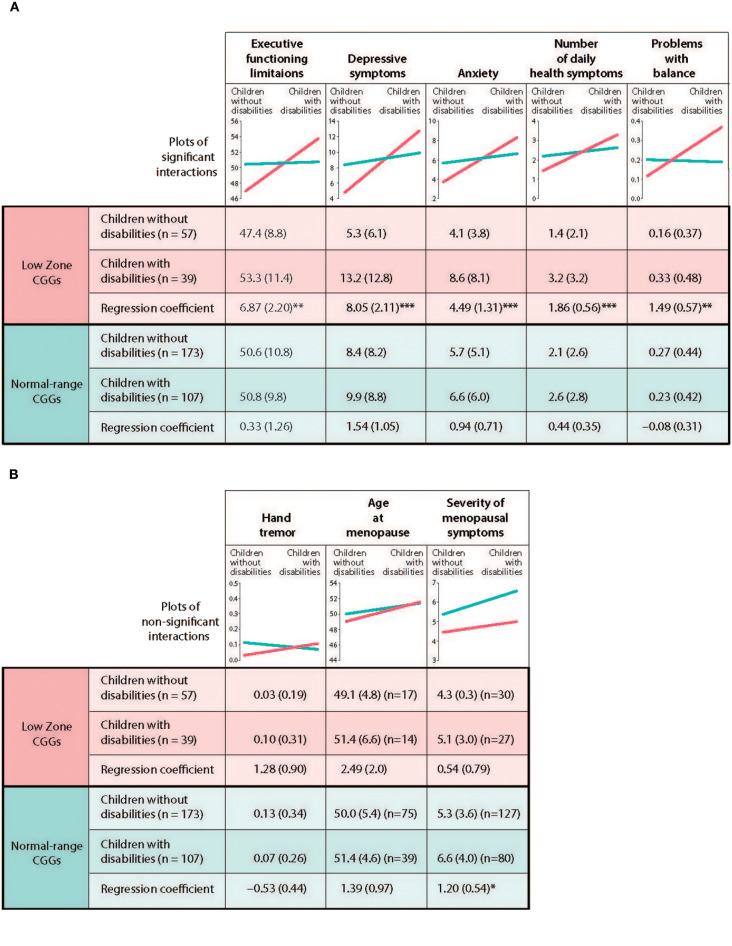
Test of differential susceptibility for mothers. **(A)** Outcomes with differential susceptibility established. **(B)** Outcomes with differential susceptibility not established. *p < 0.05, **p < 0.01, ***p < 0.001.^a^ Unadjusted means are present with standard deviations in parenthesis. Unstandardized regression coefficients are presented with standard errors in parenthesis.

Table 3 presents the results of the regression models that tested whether CGG repeat category interacted with stressful parenting status with respect to the outcome variables. The significant interaction effects are graphically displayed in Figure 2, and the means are included in Figure 3.

**Table 3:** GEE (Generalized Estimating Equations linear unless noted as logistic) models predicting phenotypes by stressful parenting and CGG repeat categories: mothers^a^.

	**Executive functioning (BRIEF-A)**		**Depressive symptoms (CES-D)**		**Anxiety (POMS)**	
	**Model 1**	**Model 2**	**Model 1**	**Model 2**	**Model 1**	**Model 2**
Maternal age	0.09 (0.04)**	0.09 (0.04)**	−0.08 (0.03)**	−0.08 (0.03)**	−0.08 (0.02)***	−0.08 (0.02)***
Number of biological children	0.38 (0.41)	0.39 (0.40)	0.11 (0.31)	0.13 (0.30)	0.26 (0.18)	0.27 (0.18)
CGG repeat categories (low zone CGGs = 1)	−0.79 (1.24)	−3.46 (1.40)*	−1.05 (1.06)	−3.67 (1.00)***	−0.59 (0.66)	−2.02 (0.62)**
Stressful parenting (parenting children with disabilities = 1)	1.96 (1.10)+	0.33 (1.26)	3.21 (0.97)**	1.55 (1.06)	1.85 (0.63)**	0.94 (0.71)
LZ x SP	–	6.54 (2.54)*	–	6.51 (2.37)**	–	3.55 (1.49)*
	**Number of daily health symptoms**		**Problems with balance (logistic)**		**Hand tremor (logistic)**	
	**Model 1**	**Model 2**	**Model 1**	**Model 2**	**Model 1**	**Model 2**
Maternal age	−0.00 (0.01)	−0.00 (0.01)	0.06 (0.01)***	0.06 (0.01)***	0.02 (0.01)+	0.02 (0.01)+
Number of biological children	0.04 (0.11)	0.05 (0.11)	−0.04 (0.10)	−0.03 (0.11)	0.07 (0.11)	0.08 (0.11)
CGG repeat categories (low zone CGGs = 1)	−0.14 (0.31)	−0.72 (0.36)*	0.08 (0.32)	−0.65 (0.45)	−0.51 (0.48)	−1.35 (0.76)
Stressful parenting (parenting children with disabilities = 1)	0.80 (0.29)**	0.43 (0.33)	0.29 (0.27)	−0.07 (0.30)	−0.18 (0.38)	−0.53 (0.44)
LZ x SP	–	1.43 (0.65)*	–	1.57 (0.63)*	–	1.81 (0.99)+
	**Age at menopause**		**Severity of menopausal symptoms**	
	**Model 1**	**Model 2**	**Model 1**	**Model 2**
Maternal age	0.05 (0.05)	0.05 (0.05)	−0.03 (0.02)	−0.03 (0.02)		
Number of biological children	−0.24 (0.40)	−0.23 (0.39)	−0.15 (0.16)	−0.15 (0.16)		
CGG Repeat categories (low zone CGGs = 1)	−0.49 (1.1)	−0.97 (1.3)	−1.22 (0.47)*	−0.92 (0.64)		
Stressful parenting (Parenting children with disabilities = 1)	1.63 (0.88)+	1.39 (0.97)	1.06 (0.46)*	1.20 (0.54)*		
LZ x SP	–	1.10 (2.22)	–	−0.66 (0.95)		

+p < 0.10, *p < 0.05, **p < 0.01, ***p < 0.001.

a *Unstandardized regression coefficients are presented with standard errors in parenthesis*.

#### Cognition

There was a significant CGG repeat category by stressful parenting status interaction effect for executive functioning, as hypothesized. Mothers in the low zone who had children with disabilities had significantly greater limitations in executive functioning than mothers in the low zone whose children did not have disabilities, whereas mothers with normal range CGG repeats did not differ significantly based on stressful parenting status.

#### Mental Health

There was a consistent pattern of interaction effects across both measures of mental health, supporting our hypothesis. Mothers in the low zone who had children with disabilities had significantly greater depressive and anxiety symptoms than low zone mothers whose children did not have disabilities. Similar to the results for executive functioning limitations, mothers with normal-range CGGs did not differ significantly in depressive or anxiety symptoms based on stressful parenting status.

#### Physical Health

Of the measures of physical health, the CGG repeat category by stressful parenting interaction was significant for the number of daily health symptoms and problems with balance. Mothers in the low zone who had children with disabilities had significantly more daily health symptoms and were more likely to have problems with balance than low zone mothers whose children were non-disabled. However, mothers with normal-range CGG repeats did not differ significantly based on stressful parenting status.

For hand tremors, severity of menopausal symptoms, and age at menopause, the interactions between CGG repeat category by stressful parenting status were not significant. However, there were significant main effects for both CGG repeat category and for stressful parenting status for severity of menopausal symptoms such that mothers in the low zone had less severe symptoms than those in the normal range, and mothers who experienced stressful parenting had more severe symptoms than those whose children did not have disabilities. Finally, there were no main effects of either CGG repeat category or stressful parenting status for hand tremor or age at menopause.

### Differential Susceptibility Analyses

We next tested the differential susceptibility hypothesis for those phenotypes for which there were significant CGG repeat category x stressful parenting status by following the five steps proposed by Belsky et al. (18). The first step is to demonstrate that there is a genuine interaction between the susceptibility factor (low zone vs. normal-range CGGs) and the predictor to show that the regression lines of the predictor for each subgroup of the susceptibility factor crossed each other. In Table 3, we showed that there were statistically significant interactions between CGG repeat category and stressful parenting status for five dependent variables. Graphic depiction of the interactions showed that the regression lines for low zone CGGs and normal-range CGGs crossed each other for these variables (see Figure 3A).

Specifically, it is not just that the effects of stressful parenting are stronger in the low zone CGG group vs. the normal-range CGG group; rather, they are weak or non-existent in the normal-range group.

The second step in establishing differential susceptibility is to demonstrate the independence of the susceptibility factor and the predictor. The partial correlation between CGG repeat category and stressful parenting status, net of maternal age and the number of children, was not significant (*r* = 0.017, *p* = 0.744), supporting differential susceptibility.

The third step in establishing differential susceptibility is to demonstrate the independence of the susceptibility factor and each outcome. Partial correlations between the CGG repeat category and each outcome, net of covariates, were not statistically significant (correlations ranging from 0.011 to 0.043 with the smallest *p*-value being 0.312), again supporting differential susceptibility.

The fourth test is to compare the regression plots with the prototypical graphs depicted in Figure 1 in the Belsky et al. (18) paper. Not only do the regression lines need to cross each other, it is required that the regression line for the susceptible subgroup (in this study, low zone CGGs) should be significantly different from zero and significantly different from that of the non-susceptible subgroup (normal-range CGGs), and further that the regression line of the non-susceptible group should not be significantly different from zero. Graphs shown in Figure 3A are consistent with the fourth criterion for differential susceptibility.

The fifth step is to test the specificity of the model by demonstrating that the current model is not replicated using other susceptibility factors and other outcomes. Regarding specificity of effects for other susceptibility factors, we were not able to test this criterion since there was no other available genetic information in the current data set. Regarding effects for other outcomes, we already showed that the interactions between CGG repeat category and stressful parenting status were not significant for hand tremor, age at menopause, and severity of menopausal symptoms (see Table 3 and Figure 3B).

### Exploring How Age Interacts With Differential Susceptibility

The wide age range of the present study participants offered the possibility of exploring whether the differential susceptibility effects detected above were stable across the mothers' life course or were more pronounced at certain stages of life. To do so, we expanded the regression models for the five outcomes where differential susceptibility had been established (i.e., executive functioning, depressive symptoms, anxiety, number of daily health symptoms, and problems with balance). These expanded regression models included all two-way interactions of the main effect variables, as well as the three-way interaction of CGG repeat category by stressful parenting by maternal age. A significant three-way interaction would suggest that differential susceptibility varied by maternal age.

**Figure 4:**
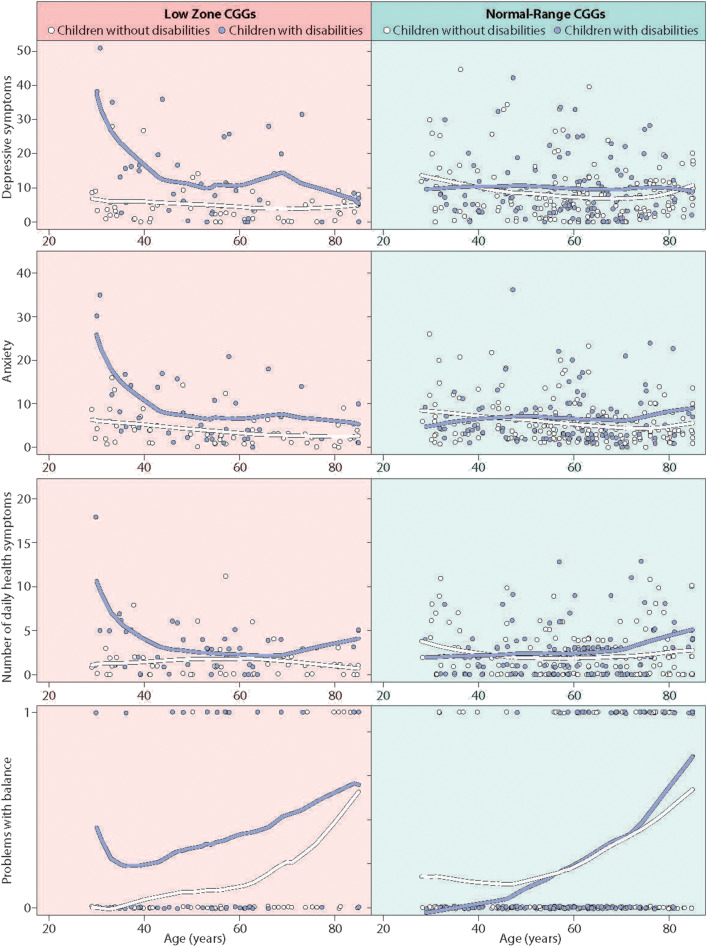
Significant three-way interaction effects of age by stressful parenting status by CGG repeat category.

The three-way interaction for executive functioning was not statistically significant; the differential susceptibility effect was similar in mothers of all ages. However, for the other four outcomes where differential susceptibility was established, maternal age was found to be a potentially important factor. Specifically, the three-way interaction effect approached statistical significance for depressive symptoms [*b* = −0.30 (0.16), *p* = 0.056], anxiety [*b* = −0.20 (0.11), *p* = 0.062], number of health symptoms [*b* = −0.10 (0.05), *p* = 0.062], and was statistically significant for problems with balance [*b* = −0.10 (0.05), *p* < 0.05]. Figure 4 presents graphs of these findings, plotting raw data (tables depicting the full models are available from the authors). As shown in the left panel of Figure 4, for mothers with low zone CGG repeats, the patterns indicate that the differential susceptibility effect was most prominent when mothers were in their 30s and 40s. At these ages, mothers in the low zone who had children with disabilities had high levels of depressive symptoms, anxiety, daily health symptoms, and problems with balance, while mothers in the low zone whose children were non-disabled had the much lower levels of these conditions. In contrast, as shown in the right panel of Figure 4, mothers in the normal range (both those who had children with disabilities and those who did not) were similar across all ages. For problems with balance, in addition to the higher level for low zone mothers at younger ages, all four groups had greater difficulties with balance at later ages.

## Results for Fathers

We conducted parallel analyses for the sample of low zone and normal-range fathers as reported above for mothers, which we report briefly here given our primary focus on mothers (all tables presenting results for fathers are available from the authors). Nearly one-third of fathers in the present sample (*n* = 181, 31.1%) had at least one child with disabilities. Fathers in the low zone and fathers with normal-range CGGs did not differ in their likelihood of having children with disabilities (30.1 vs. 31.9%, respectively), nor did they differ in the specific condition of their child. Similar to the mothers in the present sample, the most common conditions affecting the children in both CGG groups were anxiety/depression (*n* = 60) and ADD/ADHD (*n* = 36), while very few had a child with severe disabilities such as schizophrenia or autism spectrum disorder.

We conducted regression analyses that tested whether for fathers, CGG repeat category interacted with stressful parenting status with respect to the outcome variables. The regression models paralleled those conducted for mothers, and included executive functioning, depressive symptoms, anxiety symptoms, number of daily health symptoms, problems with balance, and hand tremors. Only one interaction effect was statistically significant, namely for hand tremors. Fathers in the low zone who had children with disabilities were significantly more likely to have hand tremors than those whose children with not disabled, whereas there was no such difference among fathers who had normal-range CGG repeats. Follow-up analyses indicated that this interaction effect met all criteria for differential susceptibility. However, the three-way interaction (age x CGG repeat category x stressful parenting status) was not significant.

For the other five outcome variables, there were no main effects indicating a different phenotypic pattern for fathers in the low zone and fathers with normal-range CGGs. Two main effects for stressful parenting reached statistical significance, namely number of daily health symptoms and problems with balance; fathers whose children had disabilities had a greater number of health symptoms and were more likely to have problems with balance than fathers whose children were all non-disabled. Older age was associated greater executive functioning deficits, anxiety, and problems with balance.

## Discussion

Parenting is one of the most salient and gratifying roles of adulthood and parenting a child with disabilities is surprisingly common. In the U.S., ~10% of children have ADHD (48), and there are nearly as high rates of depression or anxiety [8.4%; (47)]. Lifetime rates of these disorders are higher, consistent with the patterns observed in the present study. Studies of the impact of parenting children with disabilities report high levels of parenting stress, particularly for mothers, as well as significant heterogeneity in the degree to which parents of individuals with disabilities experience negative outcomes (23, 28, 49). In the present study, we show that variability in *FMR1* CGG repeats might be one reason why some mothers are more vulnerable in the context of parenting children with disabilities and why others may be more resilient. Specifically, in the present cohort, mothers in the low zone had greater vulnerability to stressful parenting with respect to executive functioning limitations, depressive and anxiety symptoms, health symptoms, and problems with balance. However, not all phenotypes were similarly affected; the differential susceptibility effect was not evident for age at menopause, menopausal symptoms, or hand tremors.

For mothers, this study extends our past research findings in which individuals were found to differ in their susceptibility to both positive and negative aspects of the family environment, depending on whether they have low vs. average numbers of *FMR1* CGG repeats (17). In both studies, those with low numbers of CGG repeats were more reactive to environmental stress than those with average numbers of CGG repeats; low zone mothers who had children with disabilities were more vulnerable than those whose children were non-disabled. In contrast, those with average repeat numbers were more resilient, as they did not vary in health and mental health based on the disability status of their children.

However, with the exception of hand tremors, this pattern was not evident for fathers in the present study, suggesting a more pronounced gene x environment interaction effect for mothers than for fathers. This is consistent with past research that has reported greater effects of family caregiving for mothers than for fathers (see Hayes & Watson). It is possible that different types of stress may be more salient for fathers, a possibility warranting investigation in future research.

Although these results converge with those obtained in our previous investigation using an independent population, the specific phenotypes that showed the pattern of differential susceptibility were not identical in the two studies, and these differences may be due to the inclusion of both mothers and fathers in the prior WLS study as well as to the ages of the respective study participants. In our previous WLS research, in which participants clustered tightly around 71 years of age, differential susceptibility was evident for limitations in everyday memory, number of physical health symptoms, and limitations in physical functioning, but not for depressive symptoms or anxiety. In the present research, where the participants were nearly 15 years younger on average and ranged from early adulthood to old age, a somewhat different profile of vulnerability emerged, particularly with regards to mental health.

Furthermore, in the present study, we found that it was in the earlier years of parenting (during the 30s and 40s) that mothers' differential susceptibility for depression and anxiety was most pronounced, whereas after around age 50 the groups no longer diverged. Since the participants in the WLS study were in their early 70s, it was not possible to examine differential susceptibility effects for mental health in the vulnerable age category in that study. Thus, this apparent inconsistency between the two studies may be a function of age differences between the respective participants. Although there were other methodological differences between the two studies that could have contributed to these differences in the specific phenotypes that were implicated, the overall pattern of differential susceptibility in those with low zone CGG repeats was evident in both studies.

Furthermore, in both investigations, the phenotypic characteristics of those with average numbers of CGG repeats did not differ based on whether or not they had chronic exposure to stressful parenting—this is the profile characteristic of non-susceptible genotypes. Many previous studies have noted that are substantial individual differences and great heterogeneity in response to the stress of parenting children with disabilities, with some parents manifesting profiles of resilience and others profiles of vulnerability [see (23), for a meta-analysis]. While multiple factors may contribute to this heterogeneity, our research suggests that genetics (in this case, variation in the *FMR1* gene) may be one individual difference factor.

A similar pattern of results (although with different susceptibility genes) has been shown for other aspects of the family environment. For example, Brummett et al. (50) compared women with the s/s allele of the serotonin transporter polymorphism and those with the l/l allele, and further divided the groups by whether they were caregivers for a relative with Alzheimer's or were non-caregivers. The study found that women having the s/s allele who were caregivers for a relative with Alzheimer's disease had higher levels of depressive symptoms, whereas those women with the s/s allele who were not caregivers had substantially *lower* levels of depressive symptoms than those with the l/l allele. The s/s allele conferred susceptibility to variation in the family environment. In contrast, those with the l/l allele had similar levels of depression whether caring for a relative with Alzheimer's or not. In another example, Fortuna et al. (51) compared mothers with the 7-repeat allele of DRD4-III and those without the 7-repeat, and further divided the groups into those whose infants were premature and those with full-term infants. The results showed that mothers with the 7-repeat allele manifested less sensitive parenting if their infants were preterm, but manifested more sensitive parenting if their infants were born full-term. However, those who did not carry the 7-repeat allele of DRD4-III did not differ in parenting sensitivity based on the risk level of their infants. In both of these examples, as in the present study, exposure to stressful vs. non-stressful family environments was associated with behavioral and psychological differences in genetically susceptible women, but not among women who were in the genetically non-susceptible categories for the gene under investigation.

### Limitations, Strengths, and Next Steps

The present study is limited by the use of an overwhelmingly white sample (over 90% white non-Hispanic) with measures based on responses to survey questions. Future research that includes more diverse sample members would strengthen the conclusions reported here. Details about the timing of the onset of the child's disability or the severity of the child's symptoms are not available, which would further aid interpretation of study findings. An additional limitation is the use of cross-sectional data, particularly in the investigation of age-related differences. There are likely selection effects with respect to both differential rates of survey participation and mortality among the oldest members of the PMRP population. It is also possible that there is confounding of other environmental or genetic effects that impact both the parent's health outcomes and children's risk of developmental or mental health disabilities.

Along with these limitations there are a number of study strengths, including drawing the study participants from a population-based sample and the high response rate (over 75% of eligible participants). This extension of our prior WLS research was possible due to the availability of *FMR1* CGG repeat data across the full population range, as well as measures of many of the same phenotypes. The present study's wide age range enabled extension of the past work into investigation of age effects.

Future research should probe the mechanisms that could account for the differential susceptibility observed here. Chen et al.'s (13) finding that translation is less efficient at lower numbers of CGG repeats in *FMR1* may offer a clue that can be pursued in future research, as inefficiency in translation might potentiate responses to environmental effects. Translational efficiency and/or localization of specific mRNAs are molecular mechanisms that may underlie these differences. Future studies are needed to unveil the impact of CGG repeat number of *FMR1* on neuronal function and plasticity, and to uncover the link between CGG repeat number and the genes that drive susceptibility to stress.

## Conclusions

Parenting a child with disabilities is a prevalent source of chronic stress, particularly for mothers, with substantial heterogeneity in the associated health impacts. Variation in *FMR1* CGG repeats may partially explain individual differences in resilience and vulnerability to stressful parenting. The results of the present investigation could be useful in predicting the parenting stress response in individuals with varying genotypes, and in offering resources and supports to reduce and manage high levels of stress emanating from having a child with disabilities.

Currently, there is consensus that reproducibility of results is critical to biomedical research. The largely convergent findings across two independent populations using different study designs lends credence to the idea that low numbers of CGG repeats in the *FMR1* gene may be one source of susceptibility to both the positive and negative impacts of the family environment. Studies of human populations are challenged to identify sources of individual differences, and the confluence of genetic and environmental factors provides a fruitful avenue.

## Data Availability Statement

The datasets generated for this study will not be made publicly available. The terms of the IRB protocol prohibit public sharing of the data sets. Requests to access the datasets should be directed to Marsha R. Mailick, marsha.mailick@wisc.edu.

## Author Contributions

MM, JH, and PR designed the study. JH carried out the statistical analysis. MHB provided the DNA samples. MWB carried out the assays. MM wrote the manuscript and all other authors JH, LD, JG, AM, MWB, PR, and MHB contributed to and approved the manuscript.

## Conflict of Interest

MM is the Chair of the Scientific Advisory Board of the John Merck Fund. The remaining authors declare that the research was conducted in the absence of any commercial or financial relationships that could be construed as a potential conflict of interest.
